# Supplementary material for: Heterochronic faecal transplantation boosts gut germinal centres in aged mice
Source: Nat Commun. 2019 Jun 4;10:2443. doi: 10.1038/s41467-019-10430-7 (PMC6547660; doi:10.1038/s41467-019-10430-7)
Supplement: Supplementary file 1 — Supplementary Information [file 41467_2019_10430_MOESM1_ESM.pdf]

Supplementary Information

Heterochronic faecal transplantation boosts gut germinal centres in aged mice

Stebegg et al.

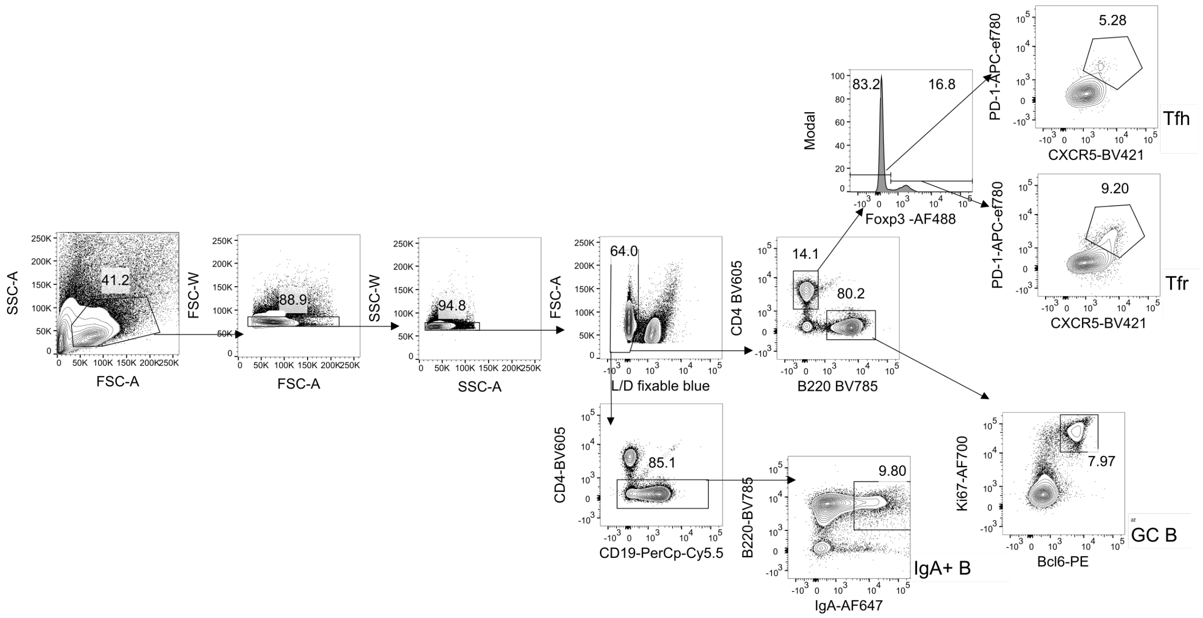

Supplementary Figure 1: Gating strategy for Tfh, Tfr, germinal centre (GC) B and IgA B cells from Peyer's patches or lymph nodes.

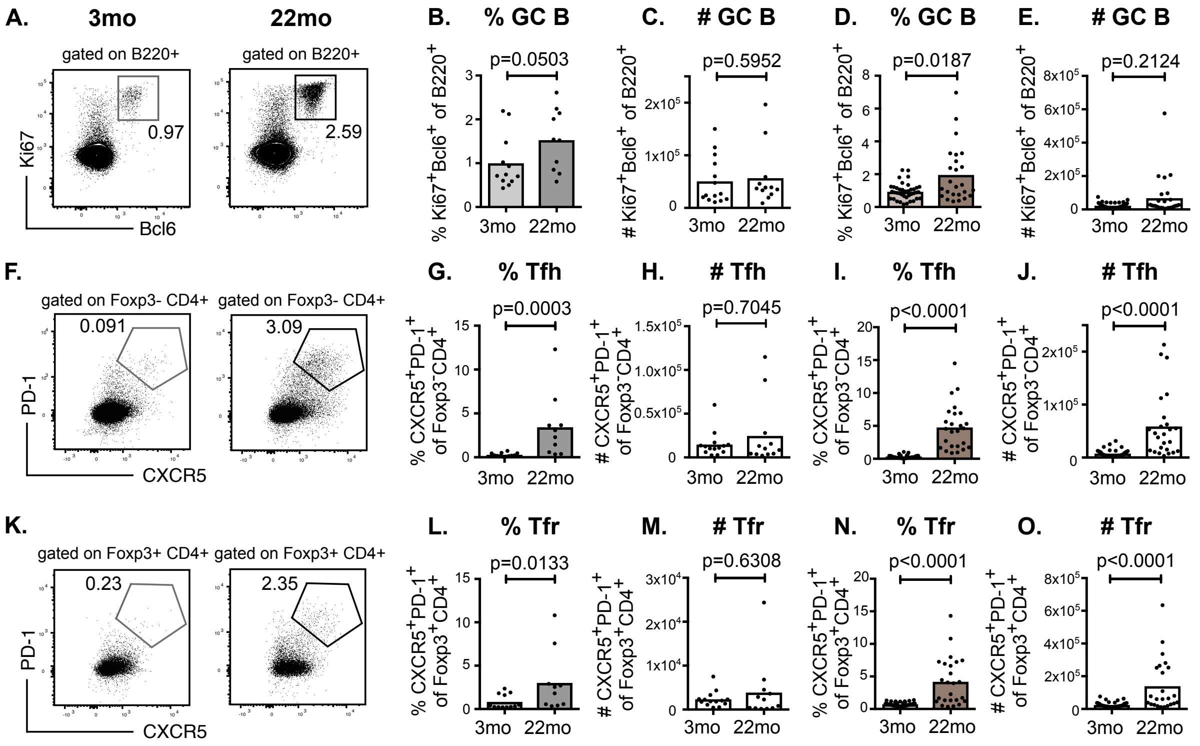

Supplementary Figure 2: Germinal centre B cells are not altered in the mesenteric lymph nodes (LNs) of aged BALB/c and C57BL/6 mice. Flow cytometric analysis of germinal centre (GC) cell populations in the mesenteric LNs of adult (3-month-old; 3mo) and aged (22-month-old; 22mo) BALBc and C57BL/6 mice. **(A-C)** Representative flow cytometric plots **(A)** and quantitation of B220+Ki67+Bcl6+ GC B cells (B, C) in the mesenteric LNs of 3-month-old and 22-month-old BALB/c mice. (D, E) Quantitation of B220+Ki67+Bcl6+ GC B cell percentage **(D)** and number **(E)** in C57BL/6 mice. **(F-H)** Representative flow plots **(F)** and quantitation of CD4+Foxp3-CXCR5+PD-1+ Tfh cells **(G, H)** in BALB/c mice. **(I, J)** Quantitation of Tfh cell percentages **(I)** and numbers **(J)** in C57BL/6 mice. **(K-M)** Representative flow plots **(K)** and quantitation of CD4+Foxp3+CXCR5+PD-1+ Tfr cells **(L, M)** in BALB/c mice. **(N, O)** Quantitation of Tfr cell percentages **(N)** and numbers **(O)** in C57BL/6 mice. Bar graphs show the combined results of 3-6 independent repeats which were performed with female BALB/c mice and both male and female C57BL/6 mice with a total of 10-41 mice per group. Bar height corresponds to the mean, and each circle represents one biological replicate. P-values were determined using the Mann-Whitney test in GraphPad Prism6. Source data are provided as a Source Data file.

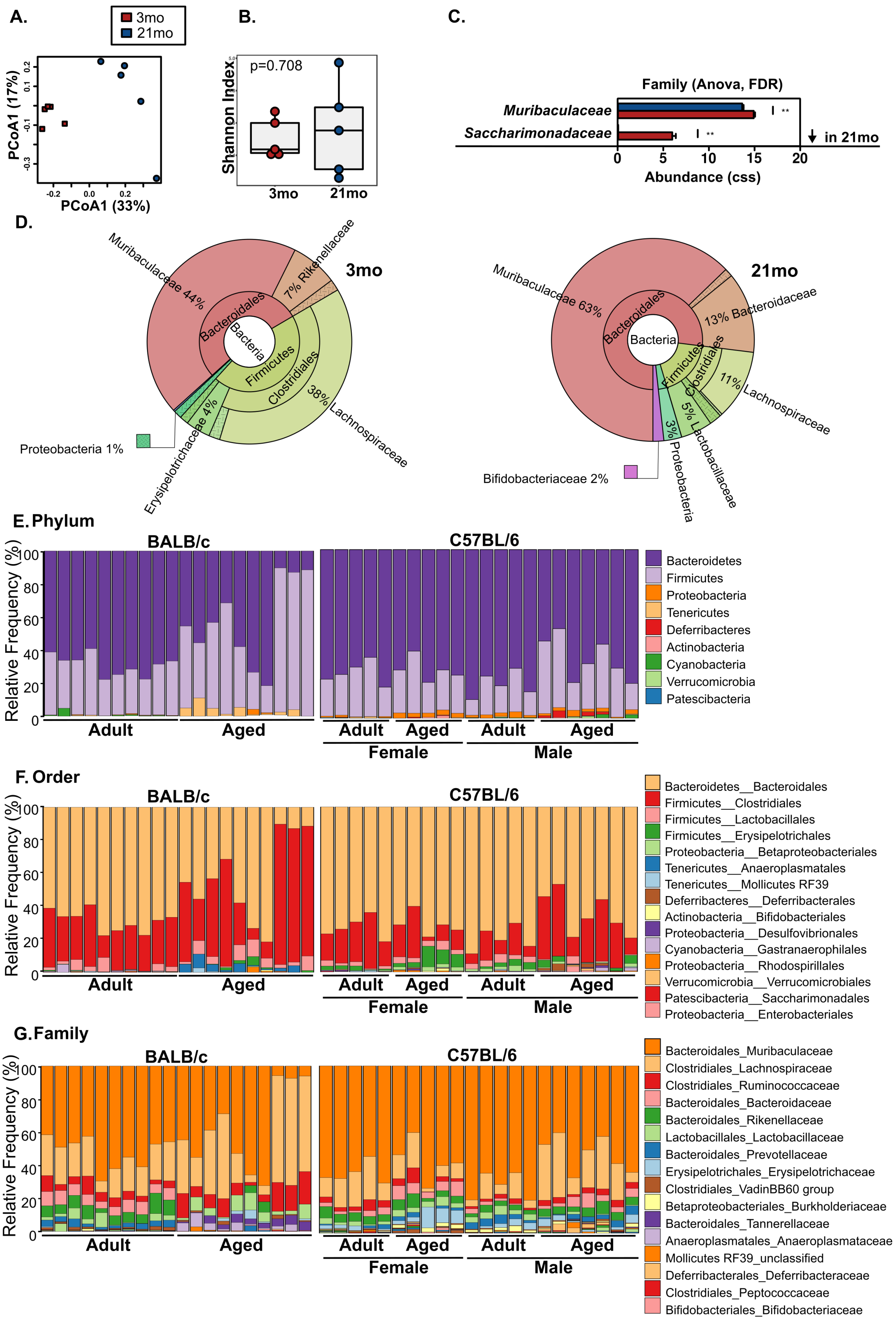

Supplementary Figure 3: Age-associated changes in the taxa composition of the gut microbiome in C57BL/6 and BALB/c mice. 16S rRNA sequencing data were generated from faecal pellets collected from five adult (3-month-old; 3mo) and five aged (21-month-old; 21mo) female BALB/c mice as well as male and female C57BL/6 mice. **(A-D)** Age-associated

changes in the gut microbiome of female C57BL/6 mice. **(A)** Bray-Curtis PCoA of samples collected from female C57BL/6 mice. **(B)** Shannon diversities of samples collected from female C57BL/6 mice. The *p*-value was generated from an ANOVA test. **(C)** Depiction of bacterial families whose abundance was significantly different between adult and aged female C57BL/6 mice as determined by ANOVA analysis after cumulative-sum scaling (CSS). *FDR ≤ 0.05, **FDR ≤ 0.01, ***FDR ≤ 0.001. **(D)** Krona plots depicting the phylogenetic composition of the gut microbiome in 3-month-old (left) and 21-month-old (right) C57BL/6 females. The percentages shown are averages of the samples in each age group. **(E-G)** Taxa plots of bacterial phyle **(E)**, orders **(F)** and families **(G)** detected in faecal samples from adult (3month-old) and aged (21-22month-old) BALB/c and C57BL/6 mice generated in QIIME2 with a total of n=5-10 mice per group. In **(F)** and **(G)** only the 15 most abundant bacterial orders/families are listed in the legend. Source data are provided as a Source Data file.

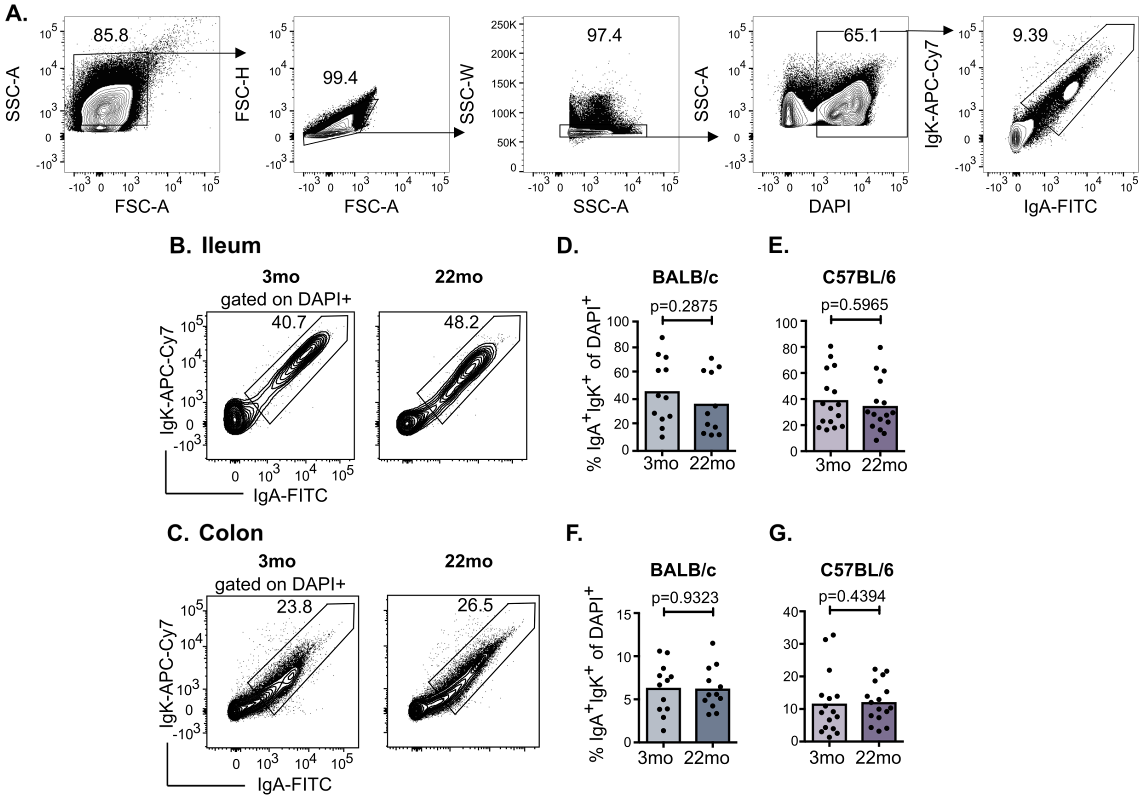

Supplementary Figure 4: IgA-coating of faecal bacteria is not affected by ageing. Bacterial IgA-coating was assessed in adult (3-month-old; 3mo) and aged (22-month-old; 22mo) mice. **(A)** Gating strategy for IgA-coated bacteria. **(B, C)** Representative flow cytometric plots of IgA-coated faecal bacteria isolated from the ileum **(B)** and colon **(C)** of BALB/c mice. **(D-G)** Quantitation of IgA-coating of bacteria in faecal contents isolated from the ileum **(D, E)** and colon **(F, G)** of adult and aged BALB/c **(D, F)** and C57BL/6 **(E, G)** mice. Bar plots show the combined results of 2-4 independent experiments with a total of n=11-16 mice per group. Bar height corresponds to the mean, and each circle represents one biological replicate. P-values were determined using the Mann-Whitney test in GraphPad Prism6. Source data are provided as a Source Data file.

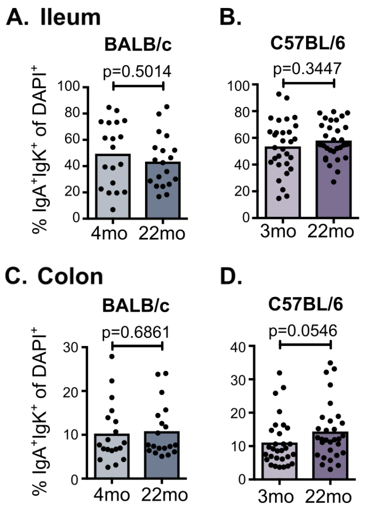

Supplementary Figure 5: Co-housing does not affect IgA-coating of faecal bacteria. Bacterial IgA-coating was assessed in adult (3-4-month-old; 3mo/4mo) and aged (22-month-old; 22mo) mice after co-housing for 40 days. **(A-D)** Quantitation of IgA-coating of bacteria in faecal contents isolated from the ileum **(A, B)** and colon **(C, D)** of cohoused BALB/c **(A, C)** and C57BL/6 **(B, D)** mice. Bar plots show the combined results of 2-4 independent experiments with a total of n=19-30 mice per group. Bar height corresponds to the mean, and each circle represents one biological replicate. P-values were determined using the Mann-Whitney test in GraphPad Prism6. Source data are provided as a Source Data file.

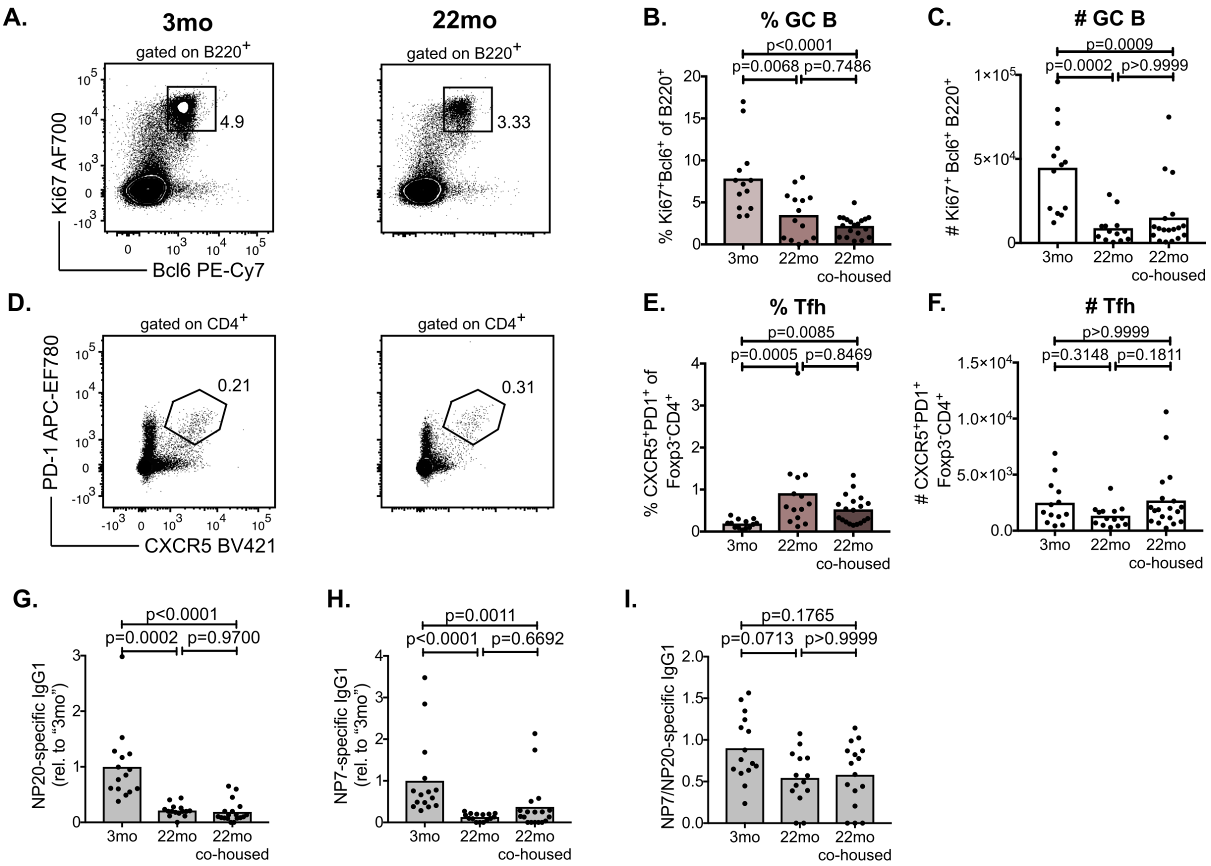

Supplementary Figure 6: Systemic immune responses are not affected by co-housing. Peripheral immune responses were analysed and compared between separately housed and co-housed adult (3-month-old; 3mo) and aged (22-month-old; 22mo) BALB/c mice on 14 days after subcutaneous immunisation with NP-KLH/Alum. **(A)** Representative flow cytometric plots of $B220^{+}Ki67^{+}Bcl6^{+}$ germinal centre (GC) B cells in the draining lymph nodes of NP-KLH-immunised mice. **(B, C)** Quantitation of $B220^{+}Ki67^{+}Bcl6^{+}$ GC B cells in percentage **(B)** and cell numbers **(C)**. **(D)** Representative flow cytometric plots of $CD4^{+}CXCR5^{+}PD\text{-}1^{+}$ Tfh cells in the draining lymph nodes. **(E, F)** Quantitation of $CD4^{+}CXCR5^{+}PD\text{-}1^{+}$ Tfh cells in percentage **(E)** and cell numbers **(F)**. **(G-I)** Affinity maturation of NP-specific IgG1 antibodies in the same mice as measured by ELISAs of NP20-specific IgG1**(G),** high-affinity NP7-specific IgG1 **(H)** and the ratio of NP7-to-NP20-specific IgG1 antibodies **(I)**. Bar plots show the combined results of two independent experiments with a total of n=13-19 mice per group. Bar height corresponds to the mean, and each circle represents one biological replicate. P-values were determined using the Kruskal-Wallis test with Dunn's multiple testing correction in GraphPad Prism6. Source data are provided as a Source Data file.

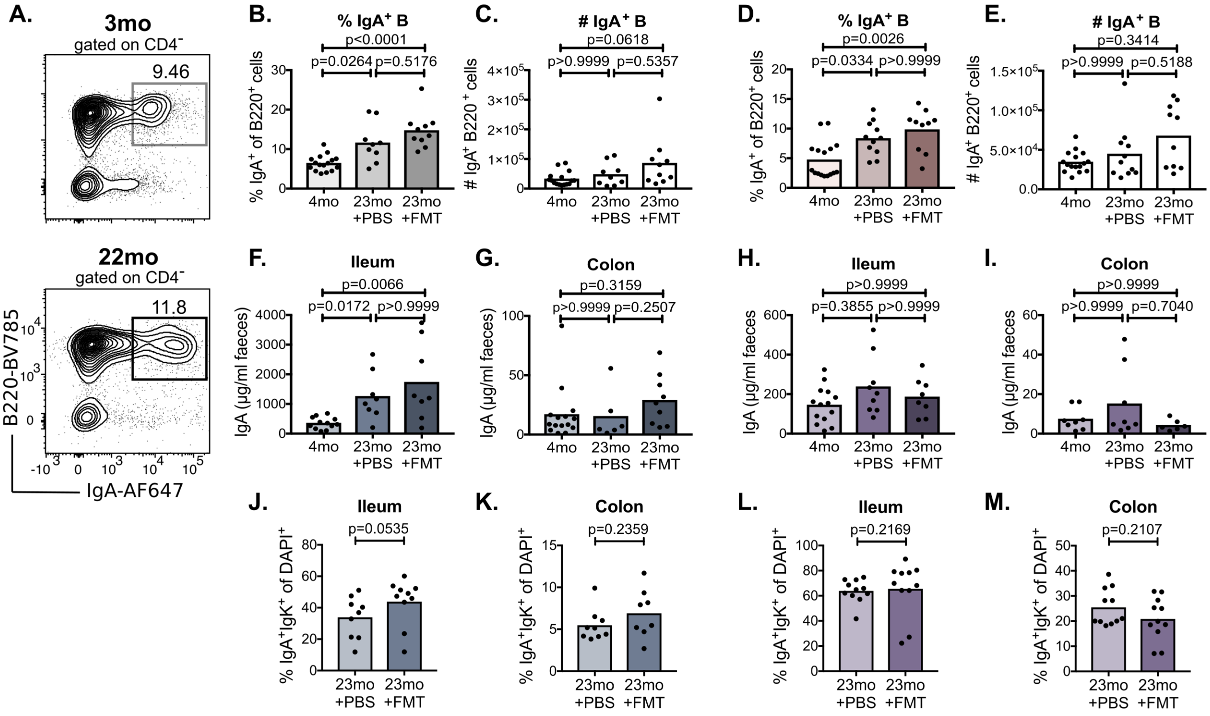

Supplementary Figure 7: Faecal microbiota transplantation (FMT) does not affect gut IgA responses. 21-month-old mice were given a suspension of faecal pellets taken from 3-month-old mice by oral gavage. The cages of these aged recipients were supplemented with fresh faecal pellets and dirty bedding from these donors once a week. A control group received PBS by oral gavage. After 3 weeks, intestinal IgA levels were assessed in adult donor mice (4mo), aged control mice (23mo+PBS) and aged mice receiving FMT (23mo+FMT). **(A)** Representative flow cytometric plots for IgA+ B cells (B220+IgA+) cells in the Peyer's patches (PPs) of adult (3-month-old; 3mo) and aged (22-month-old; 22mo) BALB/c mice. **(B-E)** Quantitation of IgA$^+$ B cells in percentage **(B, D)** and cell numbers **(C, E)** in BALB/c **(B, C)** and C57BL/6 **(D, E)** mice by flow cytometry. **(F-I)** Quantitation of faecal IgA in faecal contents isolated from the ileum **(F, H)** and colon **(G, I)** of BALB/c **(F, G)** and C57BL/6 **(H, I)** mice by ELISA. **(J-M)** Quantitation of IgA-coating of bacteria in faecal contents isolated from the ileum **(J, L)** and colon **(K, M)** of BALB/c **(J, K)** and C57BL/6 **(L, M)** mice. Bar plots show the combined results of 2 independent experiments with a total of n=8-16 mice per group. Bar height corresponds to the mean, and each dot represents one biological replicate. P-values were determined using the Kruskal-Wallis test with Dunn's multiple testing correction in GraphPad Prism6. Source data are provided as a Source Data file.
