# Supplementary material for: Heterochronic faecal transplantation boosts gut germinal centres in aged mice
Source: Nat Commun. 2019 Jun 4;10:2443. doi: 10.1038/s41467-019-10430-7 (PMC6547660; doi:10.1038/s41467-019-10430-7)
Supplement: Supplementary file 6 — Reporting Summary [file 41467_2019_10430_MOESM6_ESM.pdf]

nature research

Corresponding author(s): Michelle A Linterman

Last updated by author(s): May 7, 2019

Reporting Summary

Nature Research wishes to improve the reproducibility of the work that we publish. This form provides structure for consistency and transparency in reporting. For further information on Nature Research policies, see Authors & Referees and the Editorial Policy Checklist.

Statistics

For all statistical analyses, confirm that the following items are present in the figure legend, table legend, main text, or Methods section.

n/a	Confirmed	
☐	☒	The exact sample size (n) for each experimental group/condition, given as a discrete number and unit of measurement
☐	☒	A statement on whether measurements were taken from distinct samples or whether the same sample was measured repeatedly
☐	☒	The statistical test(s) used AND whether they are one- or two-sided<br>*Only common tests should be described solely by name; describe more complex techniques in the Methods section.*
☒	☐	A description of all covariates tested
☐	☒	A description of any assumptions or corrections, such as tests of normality and adjustment for multiple comparisons
☐	☒	A full description of the statistical parameters including central tendency (e.g. means) or other basic estimates (e.g. regression coefficient) AND variation (e.g. standard deviation) or associated estimates of uncertainty (e.g. confidence intervals)
☐	☒	For null hypothesis testing, the test statistic (e.g. F, t, r) with confidence intervals, effect sizes, degrees of freedom and P value noted<br>*Give P values as exact values whenever suitable.*
☒	☐	For Bayesian analysis, information on the choice of priors and Markov chain Monte Carlo settings
☒	☐	For hierarchical and complex designs, identification of the appropriate level for tests and full reporting of outcomes
☒	☐	Estimates of effect sizes (e.g. Cohen's d, Pearson's r), indicating how they were calculated

Our web collection on statistics for biologists contains articles on many of the points above.

Software and code

Policy information about availability of computer code

Data collection	BD FACSDIVA
Data analysis	GraphPad Prism6, FlowJo 10, QIIME2, Silva v132 SSU, Calypso v8.82-84, Metachart App

For manuscripts utilizing custom algorithms or software that are central to the research but not yet described in published literature, software must be made available to editors/reviewers. We strongly encourage code deposition in a community repository (e.g. GitHub). See the Nature Research guidelines for submitting code & software for further information.

Data

Policy information about availability of data

All manuscripts must include a data availability statement. This statement should provide the following information, where applicable:

- Accession codes, unique identifiers, or web links for publicly available datasets
- A list of figures that have associated raw data
- A description of any restrictions on data availability

The 16S rDNA sequencing data generated for this study have been deposited in ArrayExpress (E-MTAB-7750). Source data for all figures are provided with the paper as a Source Data File. Further data in support of our findings are available from the corresponding author upon request.

Field-specific reporting

Please select the one below that is the best fit for your research. If you are not sure, read the appropriate sections before making your selection.

☒ Life sciences ☐ Behavioural & social sciences ☐ Ecological, evolutionary & environmental sciences

Life sciences study design

All studies must disclose on these points even when the disclosure is negative.

Sample size	A minimum of 5 mice per group were chosen based on previous experience in the lab comparing germinal centre responses in young and aged mice. Limited availability of aged mice was a restricting factor in our study design.
Data exclusions	Some of the aged mice carried lymphomas or solid tumours which affected their immune system. Mice with visible lymphomas or large expansions of Ki67+ proliferating lymphocytes (as determined by Tukey's outlier test) were excluded from the analysis. C57BL/6 mice (especially females) were generally more affected by these symptoms than BALB/c mice.
Replication	All experiments were repeated 2-6 times. All significant changes were reproducible between experimental repeats.
Randomization	Mice were randomly allocated into age- and sex-matched experimental groups by staff of the Babraham Institute Experimental Support Unit or on the online mouse colony management system.
Blinding	Blinding was not possible due to clear/visible phenotypic differences between aged and young mice.

Reporting for specific materials, systems and methods

We require information from authors about some types of materials, experimental systems and methods used in many studies. Here, indicate whether each material, system or method listed is relevant to your study. If you are not sure if a list item applies to your research, read the appropriate section before selecting a response.

Materials & experimental systems

n/a	Involved in the study	
☐	☒	Antibodies
☒	☐	Eukaryotic cell lines
☒	☐	Palaeontology
☐	☒	Animals and other organisms
☒	☐	Human research participants
☒	☐	Clinical data

Methods

n/a	Involved in the study	
☒	☐	ChIP-seq
☐	☒	Flow cytometry
☒	☐	MRI-based neuroimaging

Antibodies

Antibodies used

PE/PE-Cy7-coupled anti-mouse Bcl6 BD Biosciences (K112-91)
APC-AF780-coupled anti-mouse PD1 eBioscience (J43)
AF488-coupled anti-mouse Foxp3 eBioscience (FJK-16S)
AF700-coupled anti-mouse Ki67 eBioscience (SolA15)
BV421-coupled anti-mouse CXCR5 Biolegend (L138D7)
BV605-coupled anti-mouse CD4 Biolegend (RM4-5)
BV785-coupled anti-mouse B220 Biolegend (RA3-6B2)
PerCp-Cy5.5-coupled anti-mouse CD44 Biolegend (IM7)
AF674/FITC-coupled anti-mouse IgA Southern Biotech (1040-02/-31)
APC-Cy-7-coupled anti-mouse IgK BD Biosciences (187.1)

Validation

All antibodies are commercially available and were validated by the manufacturers (Biolegend, eBiosciences, BD Biosciences, Southern Biotech).

Animals and other organisms

Policy information about studies involving animals; ARRIVE guidelines recommended for reporting animal research

Laboratory animals	BALB/c (only females as no aged males were available) and C57BL/6 mice (females and males) used in this study were bred and maintained in the Babraham Institute Biological Support Unit.
Wild animals	This study did not involve wild animals.
Field-collected samples	This study did not involve field-collected samples.
Ethics oversight	All mouse experimentation was approved by the Babraham Institute Animal Welfare and Ethical Review Body. Animal husbandry and experimentation complied with existing European Union and United Kingdom Home Office legislation and local standards.

Note that full information on the approval of the study protocol must also be provided in the manuscript.

Flow Cytometry

Plots

Confirm that:

☒ The axis labels state the marker and fluorochrome used (e.g. CD4-FITC).

☒ The axis scales are clearly visible. Include numbers along axes only for bottom left plot of group (a 'group' is an analysis of identical markers).

☒ All plots are contour plots with outliers or pseudocolor plots.

☒ A numerical value for number of cells or percentage (with statistics) is provided.

Methodology

Sample preparation	A single cell suspension from dissected PPs and mesenteric LNs was generated by pressing the tissues through a 70µm mesh in 2% foetal bovine serum in PBS. Cell numbers and viability were determined using a CASY TT Cell Counter (Roche). Antibody stainings of 1-3x106 cells were performed in 96 well U-bottom plates. Cells were first stained with LIVE/DEAD® Fixable Blue Dead Cell Stain (Invitrogen #L23105; diluted 1:1000 in PBS) and then incubated with FcR block for 15 minutes (anti-mouse CD16/32, clone 93, eBioscience). Surface antibody staining was performed for 60 minutes at 4oC in Brilliant Stain Buffer (BD Biosciences #563794). For intranuclear staining, cells were fixed with the eBioscience Foxp3 / Transcription Factor Staining Buffer (#00-5323-00) for 30-60min. Staining with anti-Foxp3, anti-Ki67 and anti-Bcl6 antibodies were performed for 60min at 4oC in 1x Permeabilization buffer (eBioscience #00-8333-56).<br><br>To assess IgA-coating of faecal bacteria, faecal contents were weighed and incubated for 10 min on ice in 12ul sterile-filtered PBS per mg faeces. The samples were then vortexed at full speed for 1min and spun at 500g at 4℃ for 5min to pellet bigger particles. The supernatant containing faecal bacteria was transferred to a fresh tube and spun at 10,000g at 4℃ for 5min to pellet bacteria. Bacteria were blocked in 2% bovine serum albumin (BSA) in PBS for 15 minutes, followed by staining with anti-IgA (Southern Biotech #1040-02) and anti-IgK (BD Biosciences, Clone 187.1) antibodies in 2%BSA/PBS for 45min on ice. Stained bacteria were fixed in 4% PFA overnight at 4℃. The next day, the cells were stained with DAPI (Invitrogen #D1306; 1:1000 in a staining buffer containing 0.01% Tween and 1mM EDTA) and AF594-coupled wheat germ agglutinin (Invitrogen #W11262; 1:100 in 3M KCl solution) to distinguish between gram-positive and gram-negative bacteria. The samples were acquired at 5000 events/sec on a Fortessa 5 (BD Biosciences) with the SSC threshold set to 200.
Instrument	LSRFortessa (BD Biosciences)
Software	FlowJov10
Cell population abundance	No sorts were performed for this study.
Gating strategy	The gating strategy is depicted in Supplementary Figure 1. GC B cells were defined as live/dead-B220+Ki67+Bcl6+. Tfh cells were defined as live/dead+CD4+Foxp3-CXCR5+PD1+ cells in Peyer's patches and mesenteric lymph nodes or as live/dead+CD4+CXCR5 +PD1+ cells in draining lymph nodes upon NP-KLH immunisation. Tfr cells were defined as live/dead+CD4+Foxp3+CXCR5+PD1+ cells. IgA cells were defined as live/dead-B220+IgA+ cells. IgA-coated bacteria were defined as DAPI+IgA+IgK+ events.

☒ Tick this box to confirm that a figure exemplifying the gating strategy is provided in the Supplementary Information.
